# The mechanism of eutectic modification by trace impurities

**DOI:** 10.1038/s41598-019-40455-3

**Published:** 2019-03-04

**Authors:** Saman Moniri, Xianghui Xiao, Ashwin J. Shahani

**Affiliations:** 1 Department of Chemical Engineering, University of Michigan, Ann Arbor, Michigan 48109 United States; 2 X-ray Science Division, Advanced Photon Source, Argonne National Laboratory, Argonne, Illinois 60439 United States; 3 Department of Materials Science and Engineering, University of Michigan, Ann Arbor, Michigan 48109 United States; 4 Present Address: National Synchrotron Light Source II, Brookhaven National Laboratory, Upton, New York, 11973 United States

## Abstract

In the quest toward rational design of materials, establishing direct links between the attributes of microscopic building blocks and the macroscopic performance limits of the bulk structures they comprise is essential. Building blocks of concern to the field of crystallization are the impurities, foreign ingredients that are either deliberately added to or naturally present in the growth medium. While the role of impurities has been studied extensively in various materials systems, the inherent complexity of eutectic crystallization in the presence of trace, often metallic impurities (‘eutectic modification’) remains poorly understood. In particular, the origins behind the drastic microstructural changes observed upon modification are elusive. Herein, we employ an integrated imaging approach to shed light on the influence of trace metal impurities during the growth of an irregular (faceted–non-faceted) eutectic. Our dynamic and 3D synchrotron-based X-ray imaging results reveal the markedly different microstructural and, for the first time, topological properties of the eutectic constituents that arise upon modification, not fully predicted by the existing theories. Together with *ex situ* crystallographic characterization of the fully-solidified specimen, our multi-modal study provides a unified picture of eutectic modification: The impurities selectively alter the stacking sequence of the faceted phase, thereby inhibiting its steady-state growth. Consequently, the non-faceted phase advances deeper into the melt, eventually engulfing the faceted phase in its wake. We present a quantitative topological framework to rationalize these experimental observations.

## Introduction

Impurities, whether intentionally introduced to modify a product or unavoidably present in the growth medium, have been shown to play important roles during all stages of solidification in areas as diverse as pharmaceutical and protein crystallization^1,2^, semiconductor and polymer processing^3,4^, single crystal production^5^, and process metallurgy^6^ including additive manufacturing^7–9^. In the latter case, it has been demonstrated recently that chemical modification of the feedstock alloy by impurities can vastly expand the range of compatible metallic materials that can be processed^7^, which could lead to landscape-changing advances across multiple sectors such as aerospace, automotive, and biomedical. The specificity of impurities for either inhibition, by poisoning the nucleation sites, or promotion, by lowering the barriers of nucleation, often leads to a rich variety of growth forms that are distinct from the equilibrium habit of the pure crystal.

A manufacturing process that is of both scientific interest and technological relevance is the crystallization of eutectic alloys in the presence of trace, often metallic impurities. During eutectic solidification, a parent liquid phase transforms simultaneously into two or more solid phases^10^. One important class of eutectics is the irregular eutectic, which consists of at least one faceted (*e*.*g*., Si or Ge) and one non-faceted (*e*.*g*., Al or Ag) phase. Due to the ‘stiff’ covalent bonding of the faceted phase^11^, irregular eutectics possess a non-periodic arrangement of lamellae and a non-isothermal growth front. Among these are low density Al-Si alloys of eutectic composition, which are used widely in many engineering applications, such as automotive and aerospace^12^. Ever since the discovery of eutectic Si modification in Al-Si alloys by Pacz in 1921^13^ — who incorporated a small amount of NaF (as well as separate treatments with a few other alkali halides) into the alloy melt before solidification — chemical modification as a phenomenon has been widely investigated. Various modifier species (*e*.*g*., Na, Sr, Eu, Ba, Ca, Y, Yb) have been reported to yield refinement, *i*.*e*., a change in the length scale (smaller inter-lamellar spacing), as well as favorable changes in the eutectic morphology, typically from a coarse, plate-like network into a finer, more fibrous one^14–24^. Such chemical modification is of technological interest as it improves the mechanical properties (*e*.*g*., strength and ductility) of the as-cast alloy product^12,21,25^.

A question of critical importance for the structure-property relationship of impurity-modified alloys is related to the microscopic origin of modification itself. In turn, a fundamental understanding of chemical modification will enable manufacturers to tune the complex microstructures to technological demands. Several models have been proposed since the 1960s and onwards to explain the mechanisms leading to refinement and morphological evolution in the impurity-modified alloys, but in more recent years the utilization of advanced characterization tools for *ex situ* nanoscale investigations has revealed some limitations of these classical models and shed new light on the role of impurities^19,26,27^.

Broadly, the classical models fall into one of two categories: (i) modified nucleation, or (ii) modified growth. In the former perspective, Thall and Chalmers^28^ proposed that the modifier species suppresses eutectic nucleation and shifts the eutectic composition toward higher Si content, relative to the unmodified Al-Si alloy. According to Crosley and Mondolfo^29^, the reason for this behavior might be that the modifier species poisons the Si nucleation sites, thereby causing the observed morphological changes. Additionally, these authors suggested that there is a reversal of leading roles (Al leading Si at the growth front in the modified alloy), due to a reduction in the rate of Si diffusion in the liquid in the presence of the modifying agent as well as a change in the surface tension of liquid Al. Differences in the growth rates between Al and Si results in complete encasement of Si by Al in the modified alloy, such that the Si phase is forced to continuously re-nucleate as films along the boundaries of eutectic colonies^29^. We note that Si re-nucleation would then imply a change in the topology (*i*.*e*., connectivity) of the eutectic Si, as will be expounded in detail below. In the latter (growth) perspective, the proposed models are largely based on geometric considerations of the impurity atoms and the faceted phase. The twin plane re-entrant edge (TPRE) growth mechanism describes the growth of the faceted (Si) phase facilitated by {111} Σ3 twins^30,31^. According to the poisoning of the TPRE mechanism, growth of the faceted phase is more isotropic due to selective adsorption of the modifier atoms at the twin-liquid interface (*i*.*e*., deactivating the advantage of the TPRE mechanism)^32^. In the impurity-induced twinning (IIT) mechanism, the modifier atoms adsorb instead at the {111} Si step surfaces, and the associated change in the stacking sequence facilitates the formation of frequent crystallographic twins and locally enables growth in multiple <112> directions^21^. The IIT mechanism, which assumes an FCC crystal structure for the faceted phase, projects that only the elements that meet the ‘ideal’ atomic radius ratio *r*_*i*_/*r* ∼ 1.646 (where *r*_*i*_ is the atomic radius of the impurity element, and *r* is that of the host phase) cause modification^21^. The ideal radius ratio is calculated based on the assumption that impurity atoms of appropriate size force a monolayer step to miss one regular close packed position and alter stacking sequence^21^. While the IIT model has gained some traction in the crystal growth community, several of its shortcomings have been reported, including by the original proposers^21^ and others^24,33,34^. We note that while quenching in the absence of modifying agents can produce fibrous, as opposed to flake-like, Si in Al-Si alloys, it does not change the twin density of Si^23^.

In the contemporary literature, for cases that chemical modification does result in increased twinning, the twinning mechanisms remain a subject of debate. In Eu-modified Al-Si alloys^20^, Schumacher and colleagues observed frequent twinning within eutectic Si as well as the formation of nanoscale Al_2_Si_2_Eu intermetallic clusters or continuous Eu-rich layers located ahead of the Si twins. These authors’ high-resolution scanning transmission electron microscopy results support the simultaneous occurrence of the IIT as well as poisoning of the TPRE growth mechanisms^20,21,30,31^. Additionally, the authors attribute the formation of such impurity-containing moieties as “artifact” of the adsorption and entrapment of the modifying agent within eutectic Si during growth^20^, rather than as enabler of modification as suggested previously by others^18,19^. On the other hand, in the case of Sr-modified Al-Si alloys, Kothleitner and colleagues^27^ have recently reported that prolific twinning in Si arises in the solid state, upon subsequent annealing of the as-grown eutectic microstructure. In this picture, the modifier (here Sr) atoms diffuse interstitially into the fully-solidified Si, self-organizing into <100> interstitial columns. According to these authors’ work, twin boundaries subsequently originate in the vicinity of the interstitial Sr columns.

The above and many other *post mortem* studies of fully solidified alloy samples have provided great insights into the structure and crystallography of impurity-modified irregular eutectics. However, the oft-employed ‘quench-and-look’ approach is inherently limited in scope and can only provide mechanistic inferences in limited situations. Quenching can distort the morphology of the solid-liquid interface compared to that seen during crystallization. It is also worth noting that at the typical concentrations of impurities used for chemical modification (~100 ppm), analytical methods do not possess the necessary sensitivity for conclusive determination of the local spatial distribution of the modifying element. In the contemporary viewpoints surveyed above, no *in situ* reports are available to the best of our knowledge, and no comparisons are drawn against the corresponding unmodified alloys. Evidently, a unified theory of eutectic modification — that can fully describe the microstructural and topological complexities that arise during solidification — remains elusive due to the dearth of 3D space- and time-resolved information.

In the present study, we employ an integrated imaging approach in an effort to understand the role of impurities on eutectic solidification. In particular, we examine the solidification pathways of an Al-51.6 wt.%Ge eutectic with trace metal impurities (0.1 wt.% Na) and draw comparisons with the unmodified Al-Ge alloy reported recently by Shahani and coworkers^35^. We note that Ge behaves in a similar fashion to Si discussed above, *e*.*g*., both materials have covalent bonding, high entropies of fusion, highly anisotropic Wulff shapes, and only modestly different self-diffusion coefficients at their respective melting points^36,37^. By non-destructively monitoring the solidification process *in situ via* synchrotron-based X-ray microtomography (4D XRT), we tracked the evolution of directionality and topology in the eutectic constituents for the first time. To link these dynamical events to crystallographic features, we conducted electron backscatter diffraction (EBSD) experiments on the fully-solidified specimens. On the basis of our integrated characterizations, we find that the growth of the faceted phase is retarded by the trace impurities, leading to orientational changes and topological singularities during crystallization. The resulting eutectic microstructure belongs to its own topological class, distinct from the unmodified alloy.

## Results

### Nucleation and Growth Observations

**Figure 1:**
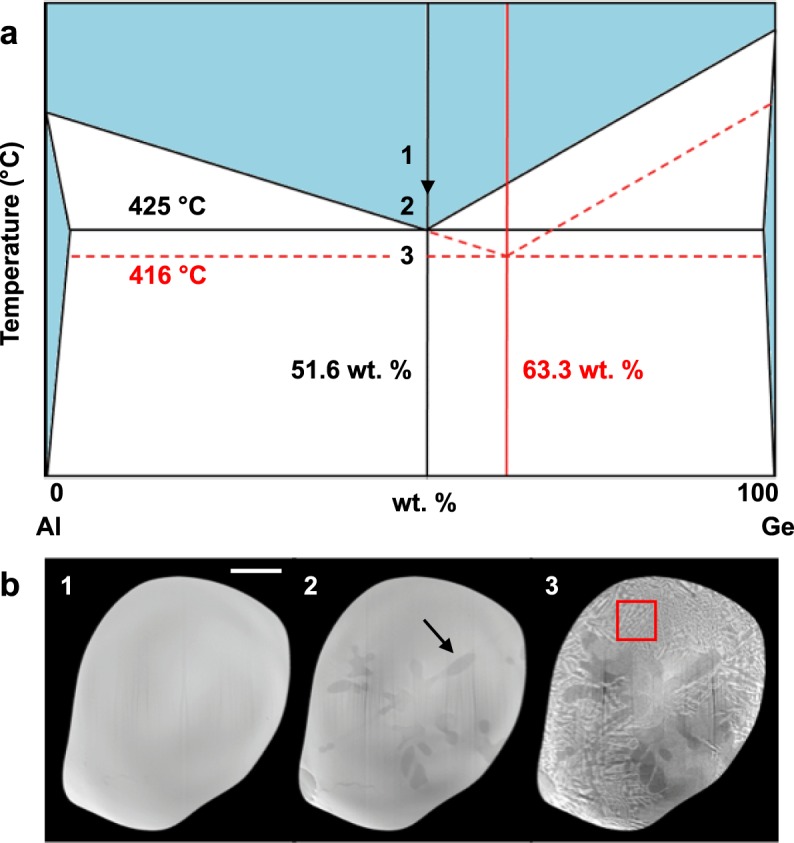
Solidification pathway. (**a**) The equilibrium phase diagram of the unmodified Al-Ge eutectic (black solid lines) is shown together with kinetic arrests in the modified Al-Ge-Na alloy (red dashed lines). The eutectic composition shifts to the Ge side (63.3 wt.%) in the modified alloy. (**b**) Representative 2D slices of the 3D reconstruction show the progression of solidification. The absorption contrast between the constituents allows distinguishing between the three coexisting phases: the liquid (light gray), eutectic Ge (white), and eutectic Al (dark gray). In panel (1) the sample is fully liquid (corresponding to region 1 in **(a)**). Upon cooling below the Al-Ge eutectic temperature (425 °C), the sample consists of primary Al dendrites in the melt, seen as dark, bulbous features in panel (2) (corresponding to point 2 in **(a)**; one marked by arrow as example). Through further cooling to 416 °C, development of the Al-Ge eutectic microstructure is observed, as in panel (3), corresponding to point 3 in **(a)**. The red box in panel (3) shows the region-of-interest isolated for subsequent analysis. Scale bar: 100 μm.

The synchrotron experiment was guided by the equilibrium phase diagram of the unmodified Al-Ge eutectic (black solid lines in Fig. 1a), with the anticipation that chemical modification leads to higher nucleation undercooling^21,28^. Initially the sample was heated in a resistive furnace to 440 °C – which is well above the eutectic temperature, 425 °C, of Al-Ge – and allowed to equilibrate. The oxide skin layer, grown by thermal oxidation, contained the molten specimen and prohibited the diffusion of the Na out of the sample bulk. Then, the sample was cooled to 417 °C and held at this temperature for 5–10 min for homogenization. Subsequently, the sample temperature was dropped down to 416 °C and was held isothermally at this temperature while X-ray projections were recorded (see Methods). In Fig. 1a, we also provide kinetic boundaries due to Na modification (red dashed lines), according to our direct experimental observations of the solidification pathway, see Fig. 1b.

We observe that primary Al (in the form of equiaxed dendrites; an example marked by the arrow in Fig. 1b) forms at ~417–418 °C and continues to grow and coarsen until a sufficiently low temperature (416 °C) is reached, at which point the nucleation of eutectic Ge in the presence of the Na modifier species occurs. Given that the primary Al phase was detected first, and immediately below the Al-Ge eutectic temperature, we expect that the Al liquidus is unaffected by chemical modification (see the oblique dashed red line in Fig. 1a); a similar argument is presented in ref.^28^ based on the analysis of cooling curves. Rather, our results indicate that it is exclusively the Ge liquidus which is depressed in the Na-modified alloy, in agreement with the observation of Hanna *et al*.^38^ In comparison, in the unmodified alloy, Al and Ge phases nucleate at the same temperature and subsequently grow in a loosely-coupled manner^35^. Ultimately, the critical undercooling for the nucleation of the modified eutectic is ~9 °C, which is approximately three times higher than that of the unmodified Al-Ge eutectic^35^. Interestingly, the nucleation of the modified Al-Ge eutectic does not occur heterogeneously on the pre-existing Al dendrites, but rather on the Al_2_O_3_ oxide skin of the sample, in agreement with *ex situ* observations of Dahle *et al*.^17^ The sample temperature was maintained at 416 °C throughout the *in situ* XRT experiment, allowing for the Al-Ge eutectic to grow with minimal undercooling.

### Tomographic Reconstructions

**Figure 2:**
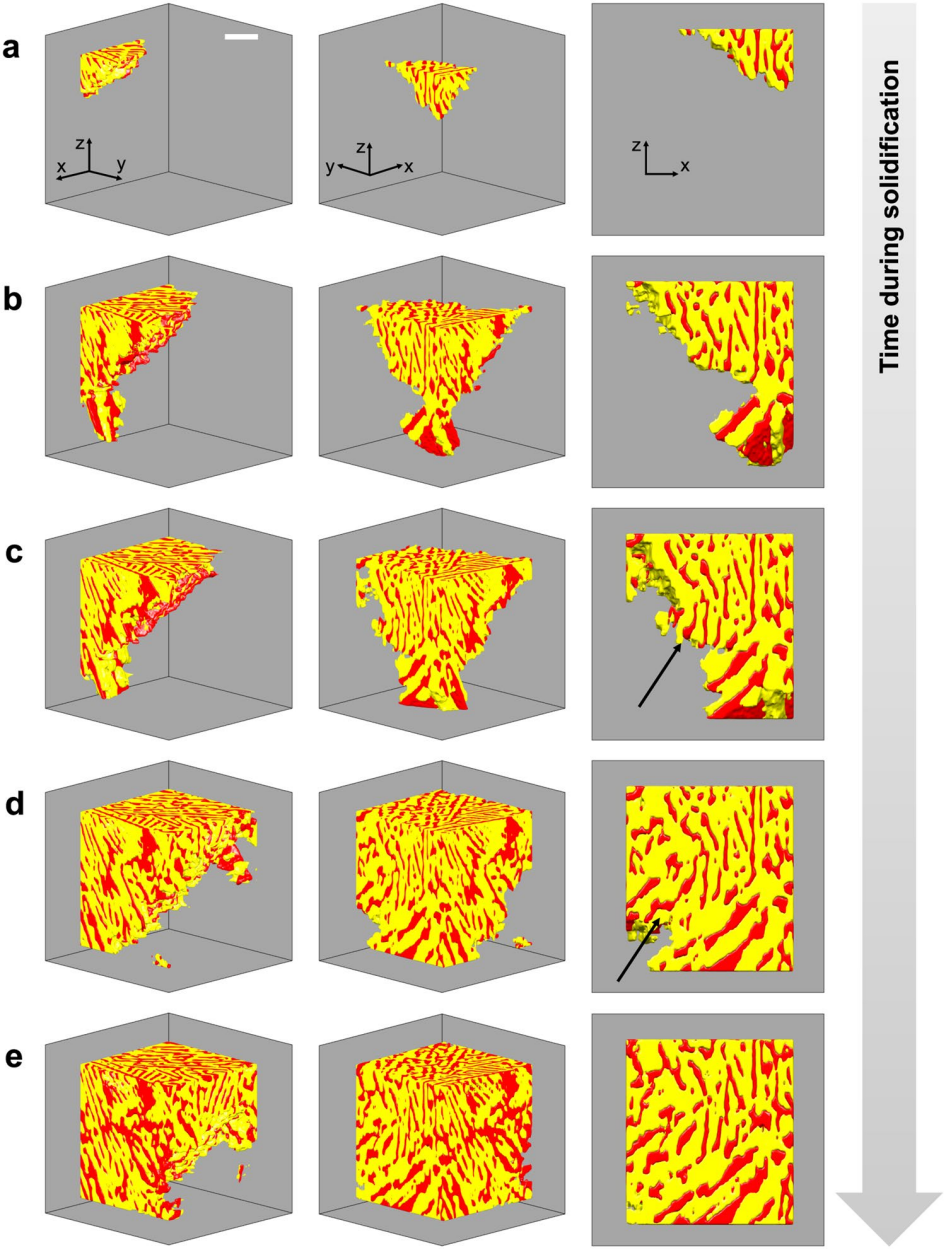
Microstructural evolution during eutectic crystallization. Three views of 3D reconstructions are shown at five representative time-steps after the start of solidification: **(a)** 180, **(b)** 220, **(c)** 240, **(d)** 280, and **(e)** 300 s. The Al phase is represented in yellow, and Ge is shown in red (the melt is rendered transparent). The arrows in the side views of **(c**,**d)** point to regions where the Al phase leads at the growth front, advancing deeper into the melt and eventually engulfing the lagging Ge phase. This morphological instability is shown to cause a marked change in the topology of the eutectic constituents. Scale bar: 100 μm.

For subsequent analysis, we analyze the dynamics of eutectic crystallization within a region-of-interest (ROI) that does not contain Al dendrites. The selected ROI provides a ‘good’ statistical representation of the eutectic microstructure because its composition (53 wt. % Ge, assessed in the fully-solidified state) matches very closely to that of the bulk alloy (51.6 wt. % Ge). A snapshot of the ROI at 260 s after the start of solidification is shown in Supplementary Fig. 1. In the 3D rendering, Ge lamellae are shown in red, and Al in yellow. The liquid phase is rendered transparent. Some Ge lamellae are observed to have highly curved solid-solid interfaces, which is anomalous for a faceted phase. As noted in Introduction, the equilibrium and kinetic shapes of semiconductor crystals tend to be fully faceted due to their strong and directional covalent bonds^36^. This curvature might arise due to the interaction of the Ge constituent with Na segregations ahead of the solidification front. To further investigate these qualitative observations, we track the growth of the eutectic region of interest in 4D. Figure 2 shows microstructural evolution during growth of the eutectic ROI at five representative time-steps. At each time-step, three views of the region are provided. Interestingly, and as indicated by the arrows in the side views of Fig. 2c,d, the Al phase leads at the growth front, and eventually envelopes the lagging Ge phase. Because the Ge phase cannot keep pace with the Al phase, steady-state growth is not possible in the modified Al-Ge system. This morphological instability is shown to cause a marked change in the topology of the eutectic constituents, which is discussed in the next section.

### Characterization of Topology

We quantify the connectivity of the eutectic constituents during the growth process by computing the topological characteristics of the microstructure^39^. In the present study, we examine three topological characteristics within the region of interest, across time: the number of independent bodies, *i*.*e*., solid ‘voids’, *v*; the genus, *g*; and the total number of loops, *i*.*e*., handles, *h*^39,40^. The genus is approximately related to the number of holes or gaps in an object: for instance, a donut or torus has a genus of one. For any object, the genus is related to the number of handles and voids according to eq. 1:

1 $$g=h-v$$

It is therefore evident that, for structures with no voids, the genus and handle have the same values. More importantly, if the number of voids exceeds the number of handles, then the genus assumes a negative value, indicating that the solid bodies are disjoint. In the context of crystallization, this situation might be encountered during the early stages of nucleation, wherein the nuclei are small and far apart. During growth the nucleated solid bodies may coalesce, giving rise to continuous handles in the microstructure and a correspondingly higher value of genus (eq. 1). In eutectic microstructures, the genera of the phases may depend on each other given the intertwined pathways available for growth.

**Figure 3:**
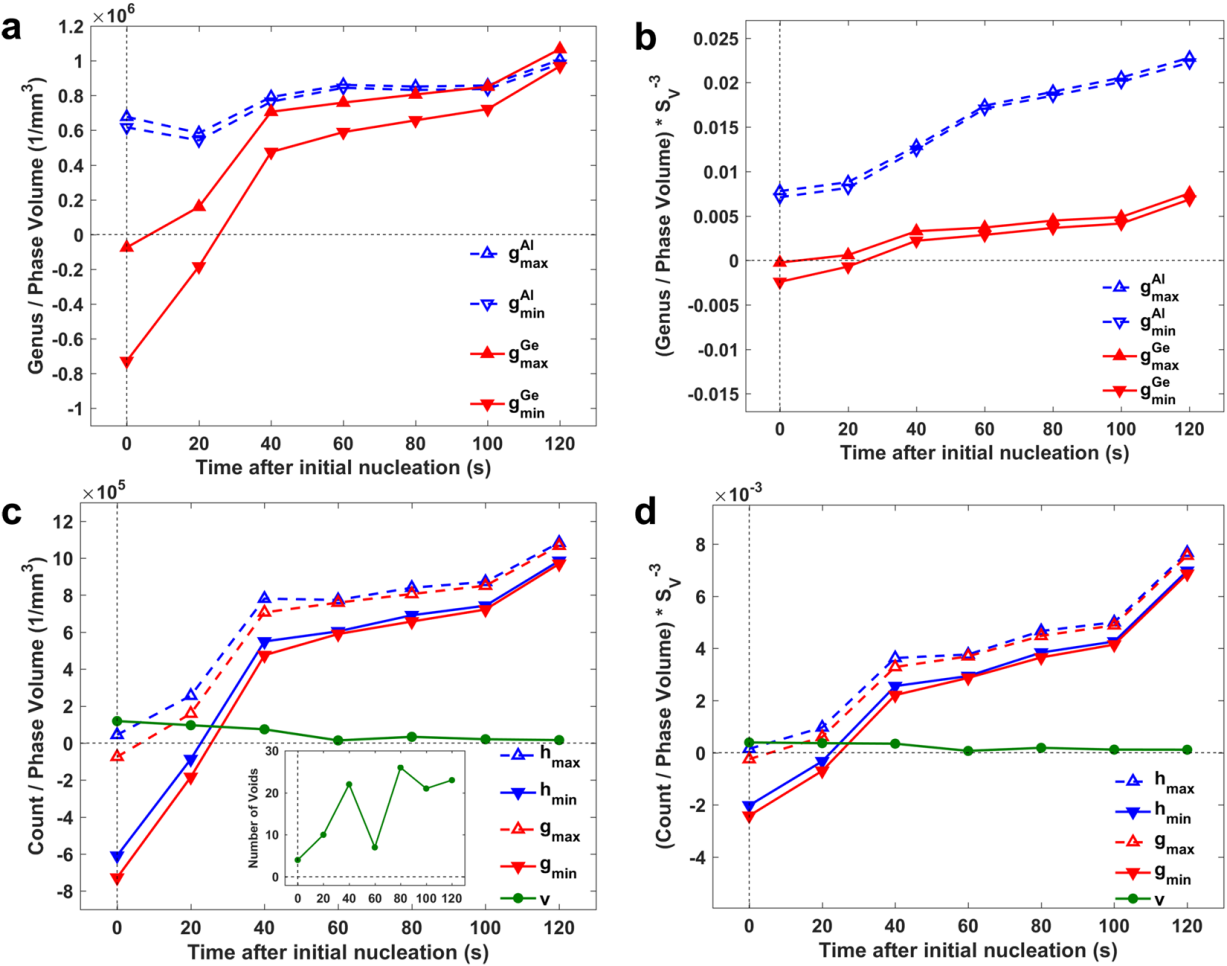
Topological distinctions between the eutectic phases. **(a)** The upper (upward arrows) and lower (downward) bounds of genus for the Al (open symbols) and the Ge (filled) phases are measured from the tomographic reconstructions. The genera are normalized by the corresponding solid phase volumes. The reference (zero) time as defined in the main text is indicated by a dashed vertical line for clarity. At this time, the genus of Ge is negative (both *g*_*max*_ and *g*_*min*_), while that of Al is positive. Therefore, the Al phase already encases the Ge phase. **(b)** The scaled (dimensionless) genera (see text) of the two eutectic constituents are displayed over time. The scaled genus of Ge is approximately an order of magnitude smaller than that of Al. **(c)** Volume-normalized topological characteristics of the eutectic Ge phase are shown, calculated by applying eq. 1 (in the text) on the mesh representation of the Ge phase. The number of solid Ge voids (inset) does not have an appreciable contribution to the topology of the microstructure, as the genus and number of handles are roughly equal. **(d)** The scaled topological characteristics of the Ge phase are displayed.

To circumvent the potential ambiguity in the calculation of genus related to the parts of the microstructure that contact the bounding box (*i*.*e*., any of six sides of the ROI), we obtain the upper and lower bounds of genus, *g*_*max*_ and *g*_*min*_, respectively^39^; computational details are provided in Methods. The true value of the genus *g* for each phase *i* is then in between the ${g}_{min}^{i}$ and ${g}_{max}^{i}$ limits. The maximum and minimum genera of Al and Ge, scaled by the corresponding solid volume, are shown in Fig. 3a. We define the reference (zero) time as the time-step during the tomographic experiment when the Ge phase is first observed (we note that this is not the precise moment of nucleation of Ge, given the 20-second temporal resolution of the tomographic experiment, nor is it necessarily the same time-step at which eutectic Al nucleates). At the reference time, several small and disjoint solid Ge domains exist. Thus, the genus of Ge is negative (both *g*_*max*_ and *g*_*min*_). We also observe that the Al phase attains a positive genus and already encases the Ge phase in a few locations (*cf*. Figure 2).

In order to eliminate the effect of the increasing length-scale of the system during the growth process, we scale the volume-normalized genus by the inverse of the surface area per unit volume^40^, ${S}_{v}^{-3}$. This scaling yields a dimensionless topological quantity $g/{S}_{v}^{-3}$ that is independent of both volume and length-scale differences of the growing microstructure over time. The dimensionless upper and lower bounds of genus for both Al and Ge are shown in Fig. 3b. While the unscaled (but volume-normalized) genus of Ge and Al have roughly the same value in the second half of the growth process, *the scaled genus of Ge is approximately an order of magnitude smaller than that of Al* (a manifestation of the greater ${S}_{v}^{-1}$ of Al compared to Ge), indicating that the eutectic Al phase engulfs the eutectic Ge phase. That is, the many holes in the Al phase (contributing to a relatively high value of ${g}_{Al}/{S}_{v}^{-3}$) are filled with independent Ge solids that are unable to grow through the solid metal layer. We note that this direct topological analysis is only possible using the 4D synchrotron data.

Full topological details of the eutectic Ge phase are shown in Fig. 3c,d. Using the upper and lower bounds for genus as defined above, together with eq. 1, upper and lower bounds for the number of handles were calculated. Figure 3c shows the evolution of the unscaled (*i*.*e*., only phase volume-normalized) characteristics during the growth process, while Fig. 3d shows the scaled (*i*.*e*., dimensionless) topological parameters. Clearly, the number of solid Ge voids does not have an appreciable contribution to the topology of the microstructure, as the genus and number of handles are roughly equal. However, it is interesting to observe that topological parameters are not necessarily monotone during the growth process. For instance, the number (absolute count) of solid Ge voids (shown in the inset in Fig. 3c) initially increases over time, followed by a sudden drop by >50% and subsequently a quick rise. In order to verify that this drop and rise in the number of voids is inherent to the data and not an artifact of the image segmentation algorithm, the connected components of the Ge phase were examined. Broadly, a connected component is defined as several interconnected structures, as opposed to a single, isolated structure^41^. As shown in Supplementary Fig. 3, immediately before the drop in the number of voids, there are several small and large Ge connected components. At the time-step at which the drop in the number of voids occurs, coalescence of those connected components is observed, justifying the decrease in the number of solid voids (fewer Ge connected components). At the following time-step, in addition to the growth of the merged connected component, several new (small) connected components (hence solid voids) are present, justifying the corresponding rise in the number of solid voids. The new Ge connected components arise due to the high undercooling (and consequently, high nucleation power) of the modified Al-Ge eutectic.

### Characterization of Directionality

**Figure 4:**
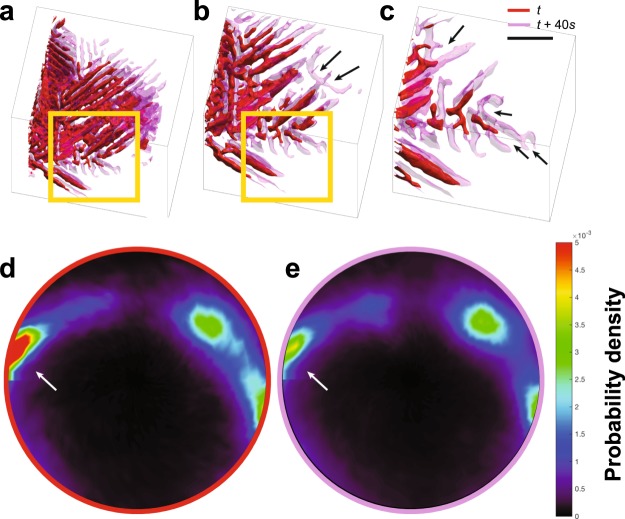
Directionality of eutectic Ge during growth. (**a**–**c**) Shown are the 3D microstructures of the Ge phase at two different time-steps, superimposed on each other. The structure at the earlier time is colored red, while that at the later time is colored pink. The two time-steps are 40 s apart. Curvature and branching events are shown with arrows in **(b**,**c**). Scale bar: 30 μm. (**d**) The interface normal distribution (IND; see text for definition) of the earlier time-step contains poles of high probability (marked by the arrow) that indicate anisotropy in growth. **(e)** The IND of the later time-step shows a diminished pole intensity compared to the earlier time-step (shown by the arrow), indicating loss of directionality during growth.

Figure 4a–c Shows the 3D microstructures of the Ge phase superimposed at two different time-steps (represented by red and pink colors). As described earlier, the *in situ* XRT results reveal significant curvature in the solid-solid interfaces of Ge, which is anomalous for the covalently-bonded faceted phase. Points of significant curvature and branching are shown with arrows in Fig. 4b,c. The degree of directionality of the microstructure is quantified by calculating stereographic projections of the orientations (normals) of patches of interface in the microstructure. Stereographic projections collapse the three-dimensional spatial orientation of the microstructure into a two-dimensional representation that facilitates the detection of any preferential directionality in the microstructure. Upon scaling the stereographic projections with the interfacial patch area, the probability of locating an interfacial patch with a particular spatial orientation is represented. We note that this so-called interface normal distribution (IND) is constructed in the reference (*i*.*e*., laboratory) frame, not the crystallographic frame. Thus, due to the many independent Ge lamellae in the bulk microstructure, the IND represents a superposition of many single crystal patterns.

The IND of the 3D microstructure of Ge for the two time-steps during growth are shown in Fig. 4d,e. The Ge facets correspond to sharp peaks in the IND, indicating a high degree of directionality of the shown lamellae. The poles of high probability present in the IND of the microstructure at earlier time-step (Fig. 4d) disappear in the later time-step (Fig. 4e), indicating loss of directionality during growth, that come about due to the curving and branching of the Ge lamellae between the two time-steps. Over time, we predict the stereographic projection to be uniformly distributed as the Ge lamellae explore the full extent of the orientation space.

### Characterization of Crystallographic Texture

**Figure 5:**
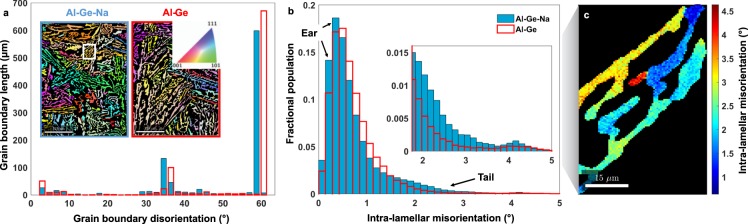
Crystallographic texture of the Ge lamellae. (**a**) Angle distribution of grain boundary disorientations (defined in main text) of eutectic Ge in both the modified (blue bars) and unmodified (red) alloys are displayed. The degree of twinning (high-probability peak at 60°) in both cases is practically equivalent. Insets show the mean crystallographic orientation of the Ge lamellae, colored according to the standard stereographic triangle in the inset. The non-indexed regions, belonging to the Al phase, are depicted as black. Scale bar: 125 μm. (**b**) Distribution of intra-lamellar misorientation of the Ge lamellae in both the modified and unmodified alloys are shown. Two features of the distributions are marked as the “ear” and the “tail”; see text for details. A significant fraction of the Ge grains in Al-Ge-Na exhibits greater intra-lamellar misorientation at higher angles (2–5°), as displayed in the inset. (**c**) Example of intra-lamellar misorientation is illustrated in a single Ge lamella of Al-Ge-Na boxed in (**a**).

Motivated by the unique directionality and topology of the eutectic Ge phase observed during the *in situ* XRT results, we investigated the individual crystallographic orientations of the fully-solidified microstructure. The microtexture (*i*.*e*., conjunction of microstructure and crystallographic texture) investigation was performed by means of electron backscatter diffraction (EBSD), on both the unmodified Al-Ge and modified Al-Ge-Na samples for sake of comparison. Analysis of a sufficiently large ROI in both cases is important to ensure statistical significance of the results. To this end, the EBSD results presented herein satisfy the convergence criterion shown in Supplementary Fig. 4. Representative orientation maps of the two alloys are shown as insets in Fig. 5a. The mean crystallographic orientation of each grain of eutectic Ge is colored according to the standard stereographic triangle on the top-right of the Al-Ge orientation map, while non-indexed regions, belonging to the Al phase, are depicted as black. The orientation maps provide only a qualitative intuition about the degree of crystallographic twinning as well as inter-lamellar spacing, both of which appear to be roughly insensitive to chemical modification by Na. Quantitative analyses of the grain boundary and intra-lamellar characteristics are discussed below.

The angle distribution of grain boundary disorientations is shown in Fig. 5a. The disorientation angle refers to the minimum of all crystallographically equivalent misorientation angles. The distribution below 2° has a value of zero, as that is the threshold used to define the boundary between two distinct orientations. For both alloys, there are obvious peaks at roughly 4°, 35°, and 60°. The near-zero peak in the angle distribution stems from the low-angle, intra-lamellar misorientation (discussed further below). The misorientation axes distribution corresponding to the two latter peaks in the angle distribution are shown in Supplementary Fig. 5. Overall, the modified and unmodified alloys exhibit similar grain boundary characteristics. The high-probability peak at 60° in the angle distribution corresponds to the <111> misorientation axes (Supplementary Fig. 5c,d). The combination 60°/<111> represents the coherent twist Σ3 grain boundary^42–44^. We observe that the degree of twinning in the unmodified and modified eutectic Ge is practically equivalent, unlike the predictions from the IIT and poisoning of TPRE models discussed above. For instance, if the twinning density were significantly higher in the modified alloy, this would suggest that IIT is operative; on the other hand, a reduction in the number of Σ3 boundaries might suggest that the re-entrant grooves were poisoned by the modifier species during crystallization.

In addition, we analyze the intra-lamellar misorientation of the Ge lamellae in both the modified and unmodified alloys, *i*.*e*., the pairwise orientation difference between points (pixels) in each Ge lamella in reference to the mean orientation of the corresponding lamella, surveyed over all lamellae inside the EBSD scan area. These results are displayed in Fig. 5b. A significant fraction of the Ge grains of the modified Al-Ge-Na displays greater intra-lamellar misorientation at higher angles (2–5°), as shown in the inset of Fig. 5b. To visualize the intra-lamellar misorientation in the Al-Ge-Na alloy, we extract the lamella boxed in the inset of Fig. 5a. This lamella was chosen since it possesses the maximum grain orientation spread (GOS), which is calculated by averaging the deviation between the orientation of each point (pixel) in a grain and the average orientation of that grain. This single Ge lamella of Al-Ge-Na is shown in Fig. 5c, illuminated according to the degree of misorientation within it. We note that a noticeable length fraction of the lamella possesses misorientation >3°, with some region of the sample having misorientation close to 5°.

Comparison of the distributions of intra-lamellar misorientation, shown in Fig. 5b, with the bivariate histogram in Supplementary Fig. 6 relating the Ge GOS and grain size in Al-Ge-Na demonstrates that the distributions differ in two important aspects, termed the “ear” and “tail” regions^45^: While the “ear” of the distribution corresponding to the modified alloy is characterized by grains of smaller size with lower intra-lamellar misorientation, the “tail” of the same distribution encompasses larger grains that have developed higher intra-lamellar misorientation. The “ear” can be explained using classical nucleation theory^28,29^: The larger nucleation undercooling of the modified eutectic (three times that of the unmodified eutectic; see above) provides a greater driving force for the formation of more Ge (and Al) nuclei per volume per time^46^. In turn, these new but smaller Ge solids do not experience the same temporal duration to grow into larger bodies that would then possess higher intra-lamellar misorientation. Meanwhile, the larger “tail” in the modified distribution reflects errors in the stacking sequence of the Ge phase during the growth process, resulting in a higher GOS; see Discussion. That is, *both* eutectic nucleation and growth are impacted in the modified alloy, as exemplified by differences in intra-lamellar misorientation.

## Discussion

It is well-established that eutectic constituents upon modification exhibit *refinement* (change in length scale)^13,15,33^ as well as favorable *morphological* changes (*e*.*g*., flake to fiber transition)^21,29,38^. Direct interrogation of the time-resolved 3D results demonstrate uniquely that chemical modification also brings about significant changes in the *topology* (*i*.*e*., connectivity) of the eutectic constituents, the reasons for which are discussed in detail below.

By monitoring the phase transformation in real-time, we found that the nucleation undercooling of the Al-Ge eutectic in the presence of Na is more than three times that of the unmodified alloy. This suggests that trace amounts of Na have a profound influence on the nucleation kinetics of the Al-Ge eutectic. In particular, primary Al dendrites nucleate prior to the faceted Ge phase, indicating that Ge is more strongly impacted than Al. Such a depression of the eutectic temperature due to chemical modification of the faceted phase is in agreement with the prediction of Thall and Chalmers^28^. Furthermore, observation of the selective affiliation of the modifier species with the faceted phase agrees with Hellawell’s prediction^22^ and is attributed to the lower activity of Na in the Al melt^47^.

**Figure 6:**
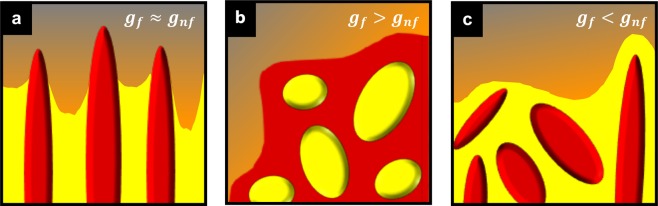
Topological classification of eutectic microstructures. (**a**) The schematic shows crystal systems with high stacking-fault energy. Due to the duplex structure of the growth front, the number of disjoint faceted components (red) would be roughly the same as the that of the non-faceted components (yellow). Thus, the genus of the faceted phase (*g*_*f*_) is comparable to that of the non-faceted phase (*g*_*nf*_). (**b**) A depiction of crystal systems with low stacking-fault energy, such as in elemental semiconductors, is shown. There, bulbs of the non-faceted phase were observed to grow through holes caused by crystallographic twinning within plates of the faceted phase^35^. In this case, the genus of faceted phase is greater than that of the non-faceted phase. (**c**) The schematic illustrates crystal systems solidified in the presence of impurities. The non-faceted phase (here Al) leads at the solidification front and eventually collapses on the faceted phase (Ge), prohibiting the latter’s steady-state propagation and resulting in a reversal of the ordering of their genera. Note that all three cases (**a**–**c**) show approximately the same volume fractions of the non-faceted (*nf*) and faceted (*f*) eutectic phases.

During growth of the modified Al-Ge eutectic, the faceted Ge phase trails behind the non-faceted Al phase. Eventually, the Ge constituent is fully engulfed in holes (gaps) within eutectic Al (Fig. 2). Therefore, growth of the Ge phase is retarded with respect to the Al phase, pointing to the influence of Na on the growth kinetics of Ge. The idea of Al enveloping Ge is consistent with previously observed overmodification bands of Al when an excess (>100 ppm) of Na is added^29,48^. The engulfment of Ge inside Al domains implies important topological behaviors of the eutectic constituents. In particular, the scaled (dimensionless) genus of Ge is about an order of magnitude lower than that of Al throughout the growth process (Fig. 3b), indicating that the Ge phase exists as independent bodies (connected components) inside holes within the Al matrix (Fig. 6c). This change of lead also has important technological significance: The brittle phase (Ge) is embedded in a ductile matrix (Al), thus contributing to the enhanced mechanical properties that have been documented previously^12^. We note that the exact opposite roles of Al and Ge (Fig. 6b) were recently observed during the growth of the unmodified Al-Ge eutectic: In the latter, the Al bulbs were observed to grow through holes caused by crystallographic twinning within the Ge plates (*i*.*e*., in-plane mosaicity); subsequently, the Al bulbs spread across the exposed Ge {111} facet planes^35^. Thus, in the unmodified microstructure, the genus of Ge would be greater than that of Al. In addition, the topology of the modified eutectic is markedly different from that expected for organic irregular eutectics, *e*.*g*., camphor-napthalene and succinonitrile-borneol systems^11^ (Fig. 6a). In this scenario, the genera of both phases would be comparable due to the duplex structure of the growth front (the number of disjoint Al components is roughly the same as the that of Ge).

Whereas eutectics have been traditionally classified in terms of entropies of fusion and constitutive volume fractions^49^, we suggest an alternative classification based on the relative magnitudes of the phase topologies. In our schema, there are three topological classes for eutectic microstructures, that come about due to variations in alloy composition and defect density. For example, when defect densities and impurity concentrations are low, one might expect to see the classical picture shown in Fig. 6a. On the other hand, in low stacking-fault-energy semiconductors, twinning is rampant (especially at high growth rates^21,50^), and one might instead obtain a microstructure akin to Fig. 6b. Lastly, when impurities selectively modify the growth kinetics of the faceted phase, the most probable scenario is that of Fig. 6c. More broadly, the higher degrees of freedom in the univariate eutectic reaction (*i*.*e*., three components and two solid phases) bring about morphological and topological transitions that are not seen in nonvariant eutectics^51^. *Thus*, *interfacial topology represents a signature of the structural complexity in each case*.

The reason for this remarkable topological modification remains to be determined. While our observation of Al leading and encasing the Ge at the growth front agrees with studies done by Thall and Chalmers and Crosley and Mondolfo^28,29^, we propose that the root cause of this effect is not the ‘re-nucleation’ of Ge. If that were the case, the introduction of the new independent bodies, *i*.*e*., solid voids, of Ge would cause a decrease in the genus of Ge (eq. 1). However, in the present study the genus of Ge is observed to increase over time (Fig. 3). Instead, the most compelling reason for the retardation of the growth of Ge is that Na interferes with the stacking sequence of Ge, leading to the greater intra-lamellar misorientation of Ge, as we show in Fig. 5b. This intra-lamellar misorientation in turn facilitates the development of curved solid-solid interfaces, enabling the Ge phase to grow without ever attaining its fully-faceted shape. Importantly, Na does not satisfy the geometric condition of Hellawell’s IIT mechanism^21^ nor increase the twinning frequency in Ge (Fig. 5a). Yet, as discussed above, Na modification is demonstrated to cause a marked change in both the morphology and the topology of the eutectic constituents. It is therefore evident that the conventional growth mechanisms of IIT, TPRE, as well as poisoning of TPRE are limited in scope. The former model is overly deterministic in that it assumes that the impurity atoms fit perfectly in the Ge lattice so as to create coherent Σ3 twin defects^21^. In reality, adsorbed impurities can give rise to a *range* of interfacial configurations, grain orientations, and misorientation angles beyond 60°.

Our results support and generalize the chemical modification model proposed by Schumacher and colleagues^20,26,33^, wherein the segregation and/or adsorption and subsequent entrapment of the impurity species (here Na) during growth of the faceted (here Ge) phase lead to modification. In this picture, the Na and Al segregate ahead of the growing Ge phase (where the partition coefficients *k*_*Na*_ < 1 and *k*_*Al*_ < 1, respectively); then, during continuous Ge growth, the adsorption of Na atoms on the exposed Ge facets may occur, forcing a change in the stacking sequence of the faceted phase at the growth front. Total-energy minimization calculations, as well as vibrational spectroscopy results, show that the adsorption of Na on Ge surfaces does give rise to new vibrational states, forming adatom-surface bonds^52^. Finally, the overgrowth of Ge leads to a solute entrapment within the eutectic Ge phase^20,26^. We note that *entrapment* should not be confused with *solute trapping*, wherein solute atoms are “frozen” in the liquid state. Given that our *in situ* tomographic experiment was conducted with minimal undercooling, solidification rates (~1 μm/s) are not high enough to physically trap the trace impurity atoms.

The aforementioned solutal entrapment likely forms an aggregation of Al-Ge-Na within, as well as in the vicinity of, the eutectic Ge phase. In the present study, we aimed to map the atomic distribution of Na in the fully-solidified specimen using high-resolution (scanning) transmission electron microscopy together with energy-dispersive spectroscopy as well as electron energy loss spectroscopy. However, our chemical mapping efforts did not yield conclusive insights, due to the high overlap of the peak from Ge L_2,3_ absorption edge (1217–1248 eV) and the small peak from the Na K-edge (1072 eV), given the trace atomic content of the latter (<0.2 at. %), in the HR-STEM images. Continuing advances in the design of analytical tools for high-resolution characterization of materials containing light elements as well as those prone to beam irradiation, will aid further analysis of the atomic distribution of Na in future experiments.

In summary, by employing a multi-modal imaging approach, we tracked the growth behavior of a modified irregular eutectic alloy and analyzed the physical characteristics of the fully-solidified specimen. We illustrated that the trace presence of the modifier species has a profound influence on the kinetics of both nucleation, as manifested in higher undercooling compared to the unmodified alloy, and growth, as expressed in the marked difference in the morphological and topological properties. These trends can be rationalized by noting that the impurity species selectively alters the stacking sequence of the faceted phase, as indicated by the higher intra-lamellar misorientation of the faceted phase in the impurity-modified alloy (over a two-fold increase). This result supports the notion that during growth, the faceted phase rejects the modifier atoms; subsequently, the modifier species adsorbs onto or in the vicinity of the faceted phase, thereby acting as “obstacles” to its continued propagation. Consequently, the non-faceted phase supersedes the faceted phase during growth, leading to a topological transition (complete encasement of the faceted phase). Collectively, our findings demonstrate a comprehensive understanding of the role of trace impurities during eutectic crystallization.

Although our results concern eutectic systems, nothing precludes the immediate extension of our techniques to materials classes that involve the interaction of impurities and other building blocks. Exploiting analogies with interactions among impurities and other building blocks, aided by powerful computational schemes, can help derive design rules that span across a vast array of materials systems. As an example, the newly investigated Al-Ce alloys demonstrate castability, structure, and mechanical strength similar to the near-eutectic Al-Si alloys modified by Sr; as a result, the Al-Ce alloys have been rated positively for laser additive manufacturing (no cracking or porosity)^53^. It is reasonable to assume that chemical modification, a lesson from a much older manufacturing process, namely casting, will provide unique pathways toward future design of alloys – including Al-Ce – by additive manufacturing.

## Methods

### Experimental Design

An integrated imaging approach, comprised of 4D (3D space- and time-resolved) synchrotron X-ray microtomography (4D XRT) was employed to directly observe the solidification pathways and interfacial dynamics of an irregular eutectic system in the presence of trace metallic impurities. We illustrate our findings on the Al-51.6 wt.%Ge eutectic with 0.1 wt.% Na as the impurity species (‘modifier’). Direct comparisons are drawn against the unmodified Al-Ge alloy reported recently^35^. Microstructural and topological evolution as measured in the reconstructed volumes are linked to the crystallographic features of post-growth samples using electron backscatter diffraction.

### Sample Preparation

Alloy buttons of nominal composition Al-51.6 wt.%Ge-0.1 wt.%Na were cast *via* vacuum arc-remelting at the Materials Preparation Center at Ames Laboratory (Ames, IA, USA), using 99.999% purity Al, 99.999% purity Ge, and >99.9% purity Na. We note that the composition of Ge (51.6 wt.%) in the present study is the same as that in the recent report of the unmodified, fully eutectic alloy investigated in ref.^35^. For the synchrotron X-ray tomography (XRT) experiments, the as-prepared alloy buttons were cut in the shape of cylindrical rods of 1 mm diameter by 5 mm length *via* electrical discharge machining.

### Beamline Setup

The XRT experiments were conducted at Sector 2-BM at the Advanced Photon Source in Argonne National Laboratory (Argonne, IL, USA). The polychromatic ‘pink’ X-ray beam was focused on the samples and a 20 μm thick LuAg:Ce scintillator converted the transmitted X-rays to visible light. High-resolution imaging was accomplished utilizing a PCO Edge CMOS camera equipped with a 10x magnifying objective to provide isotropic pixel sizes of 0.65 mm × 0.65 mm. The tomographic field-of-view measured 2,560 × 600 pixels (i.e., 1,664 mm in width by 390 mm in height). The camera frame rate and exposure time were 50 Hz and 14 ms, respectively. Due to the small penetration depth through the ‘heavy’ element Ge (51.6 wt.%), relatively long exposure times were required to ensure high signal-to-noise images. Given the 1 mm diameter of each sample, the temperature distribution was assumed to be uniform within the sample. Our beamline setup follows closely that of ref.^35^. During acquisition, the sample was rotated continuously at a rate of 6° per second. During each 180° rotation of the sample, 1,500 projections were collected. The large number of projections recorded (in addition to the high exposure time of 14 ms) guaranteed high-quality images. This combination of acquisition parameters optimally allowed for a temporal discretization of 20 s between consecutive 3D reconstructions. Data were collected for roughly 450 s, resulting in 22,500 total projections and 15 total reconstructions.

### Data Visualization

Reconstruction of the tomographic data was performed using TomoPy, a Python-based open source framework^54^. Within TomoPy, the X-ray projections were first normalized by the dark- and white-field images to account for beam instabilities. Additional correction for “ring” artefacts was made *via* combined wavelet-Fourier filtering^55^. Subsequently, the data were reconstructed *via* the direct Fourier-based Gridrec algorithm^56^. The reader is referred to ref.^54^, and references therein, for further details on these algorithms. A representative grayscale 2D slice of 3D reconstruction, parallel to the axis of rotation of the cylinder-like sample, is given in Supplementary Fig. 1a. The strong absorption contrast between the constituents allows one to easily distinguish between the three coexisting phases: eutectic Ge (white), eutectic Al (dark gray), and the liquid (light gray). The reconstructed image also features bulbous Al dendrites (also dark gray). One eutectic region free of dendrites (Supplementary Fig. 1b) is boxed and isolated for subsequent analysis. In order to enhance the contrast between the solid and liquid phases, and minimize any systematic image artefacts, reconstructions of the fully-liquefied sample were subtracted from all other reconstructions. Subsequently, the grayscale reconstructions of this region were segmented, *i*.*e*., transformed into a computable representation of their parts (liquid, eutectic Ge, and eutectic Al). Our segmentation algorithm, implemented in the Image Processing Toolbox™ of MATLAB R2016a, comprised of (i) multi-level thresholding, (ii) edge-based methods for finding the boundaries of ‘objects’ (Ge and Al) within images, and (iii) morphology-based methods (*e*.*g*., dilation and erosion) in order to remove speckle noise (small objects) as well as to smooth the border of large objects^57^. Figure S1C shows the segmentation output of the region in Supplementary Fig. 1b, wherein eutectic Ge is indicated with white, eutectic Al with gray, and air (pore) with black. In Supplementary Fig. 1d, edges of the segmentation output are overlaid on the original grayscale reconstruction (edges of Ge are red, and those of Al are yellow), showing excellent agreement with the eutectic structures underneath. The segmented 2D images were then stacked along the third spatial dimension to reveal the 3D microstructures. Supplementary Fig. 1e shows the 3D eutectic structure corresponding to the region of interest (boxed). The slice shown in Supplementary Fig. 1a corresponds to the top-most surface of the 3D visualization in Supplementary Fig. 1e.

For subsequent (topological and directional) analysis of the 3D microstructures, we represented the interphase interfaces using a triangular mesh, wherein sequences of vertices and triangular faces comprise the solid-liquid and solid-solid interfaces. To remove any ‘staircase’ artifacts, the triangular meshes were smoothened *via* mean curvature flow^58^. The 3D visualization in Supplementary Fig. 1e is an example of the smoothed structure (triangular faces and vertices are rendered colorless).

### Topological Calculations

The topological parameters were calculated from the triangular mesh representation of the segmented interphase interfaces as an input. The mesh consists of *n* nodes, *e* edges, and *f* faces. In these calculations, the genus *g* was computed according to eq. 2:

2 $$g=1-\frac{\chi }{2}$$

where

3 $$\chi =n-e+f$$

*χ* is the Euler characteristic, which relates the number of nodes (*n*), edges (*e*), and faces (*f*) of a polygonal mesh to the genus *g* of the underlying object^59^. Since the genus is a topologically-invariant property, it does not depend on the fineness or smoothness of the mesh representation of the object. Subsequently, eq. 1 (main text) was used to compute the contributions of handles *h* and voids *v* to the genus *g*. We computed and report upper and lower bounds for genus, *g*_*max*_ and *g*_*min*_, respectively. These two distinct boundary conditions are determined by treating the parts of the microstructure that contact the bounding box. To obtain *g*_*max*_, we assume that all such parts of the microstructures cross the boundary and connect to an external node, as illustrated in Supplementary Fig. 2a. To obtain *g*_*min*_, we assume that those parts of the microstructure are ‘capped’ at the bounding box, as shown in Supplementary Fig. 2b, so that there is no point of contact between the microstructure and the bounding box. In our digital (mesh) representation of the 3D microstructures, the latter is accomplished by padding all six sides of the bounding box by arrays of zeros.

### *Ex situ* Characterization

The XRT experiment was complemented with *ex situ* characterization studies. Crystallographic investigation of the as-solidified Al-Ge and Al-Ge-Na eutectics by means of electron backscatter diffraction (EBSD) was performed using the field-emission gun scanning electron microscope Tescan MIRA3 at the University of Michigan campus. For this purpose, a small cut of the high-purity alloy button was thermally annealed at 550 °C for 4 hours, and mechanically ground and polished so as to obtain scratch-free surface. For the EBSD experiments, the beam voltage was adjusted to 10 kV; the working distance, tilt, and step size were set to 20 mm, 70° relative to normal incidence, and 0.5 μm, respectively. Analysis of the texture data was performed using the MATLAB toolbox MTEX^60^ in order to generate orientation maps and misorientation distributions.

## Supplementary information

Supplementary Information

## Data Availability

All processed data needed to evaluate the conclusions in the paper are present in the paper and/or the Supplementary Materials. The raw XRT projection data are publicly available in the University of Michigan Deep Blue Data repository at 10.7302/Z2154F89.
