# Supplementary material for: The mechanism of eutectic modification by trace impurities
Source: Sci Rep. 2019 Mar 4;9:3381. doi: 10.1038/s41598-019-40455-3 (PMC6399325; doi:10.1038/s41598-019-40455-3)
Supplement: Supplementary file 1 — Supplementary Information [file 41598_2019_40455_MOESM1_ESM.docx]

Supplementary Information for

**The mechanism of eutectic modification by trace impurities**

Saman Moniri, Xianghui Xiao, and Ashwin J. Shahani

**Supplementary figures**

**Supplementary Figure 1| Segmentation and visualization of tomographic data. (a)** A representative (“raw” grayscale, 260 s after the start of solidification) 2D slice of the 3D reconstruction serves as the input for computer vision algorithm. For subsequent analysis, a region‑of‑interest (green box) was extracted. Scale bar: 200 μm. **(b)** A magnified version of the region-of-interest is shown. The eutectic Ge lamellae appear as brighter features while the eutectic Al phase appears as darker gray. Scale bar: 50 μm. **(c)** Segmented (output) image corresponding to the same “raw” (grayscale) image in **(b)**. The eutectic Ge phase is shown as white, and the eutectic Al is displayed gray. **(d)** The edges of the segmentation output are overlaid on top of the raw (grayscale) image. Edges of eutectic Ge are red, and those of eutectic Al are yellow. The segmentation result shows great agreement with the structures underneath. **(e)** Three-dimensional reconstruction of the region-of-interest at this particular time-step. The eutectic Ge phase is shown in red, Al in yellow. The melt is rendered transparent. The 2D slice in **(a)** corresponds to the top side of the 3D reconstruction in **(e)**. Scale bar: 200 μm.

**Supplementary Figure 2 | Boundary conditions for genus calculations. (a)** The parts of the solid phase that contact the bounding box of the ROI are assumed to cross it and meet at an external node. This assumption provides an upper bound for the genus ($g_{max}$) of the solid phase. **(b)** The parts of the solid phase that would contact the bounding box are capped, so that there is no longer a point of contact between the two. This configuration yields a lower bound of the genus ($g_{min}$) of the solid phase. Figure adapted from ref.^1^ with permission from John Wiley and Sons.

**Supplementary Figure 3 | Coalescence of Ge lamellae during the growth process. (a)** Several solid voids (connected components) of eutectic Ge phase are present, varying from small to large sizes. The solid voids are illuminated by different colors so that no two neighboring connected components have the same color. Scale bar: 200 μm. **(b)** Upon growth, the Ge lamellae coalesce, reducing the number of solid Ge voids, as observed in the inset of Fig. 3c (in the main text). The arrows point to coalescence events of the Ge connected components. **(c)** The coalesced Ge voids (connected components) grow while new, smaller solid voids appear, giving to the rise in the total number of solid Ge voids (see the inset of Fig. 3c in the main text).

**Supplementary Figure 4 | EBSD convergence tests.** The length fraction of Σ3 grain boundary (G.B.) as a function of EBSD scan area was used as the metric of statistical convergence. Convergence was achieved for a minimum scan area of 280 μm $\times$ 280 μm (indicated by the vertical dashed line in figure) for both Al-Ge-Na and Al-Ge EBSD patterns, as the Σ3 length fraction for each alloy reached its steady-state value. All EBSD results reported in the main text satisfy this convergence condition.

**Supplementary Figure 5 | Disorientation axis distributions corresponding to peaks in angle distributions. (a)** The axis distribution of 35° misorientation in Al‑Ge‑Na. The combination 35°/<101> corresponds to the Σ9 coincident site lattice (CSL); see Supplementary Note 1. **(b)** The axis distribution of 35° misorientation in Al-Ge. **(c)** The axis distribution of 60° misorientation in Al‑Ge‑Na. The combination 60°/<111> corresponds to the Σ3 CSL. **(d)** The axis distribution of 60° misorientation in Al‑Ge. No significant differences are observed in the axis distribution of misorientation in the modified and unmodified alloys.

**Supplementary Figure 6 | Grain size *vs*. orientation spread of eutectic Ge in Al-Ge-Na.** The bivariate histogram relating the Ge grain orientation spread (defined in the main text) and grain size in Al‑Ge‑Na demonstrates that smaller Ge grains tend to possess smaller grain orientation spreads. The data can be used to interpret the trends in the intra-lamellar misorientation shown in Fig. 5b in the main text.

**Supplementary Note 1 | Angle‑axis pairs of misorientation (accompanying Supplementary Figure 5)**

In the misorientation axes distribution shown in Supplementary Figure 5 (a and b) corresponding to the 35° misorientation angle, minor peaks are present at the <110> axes of the Al‑Ge‑Na sample, which are absent in unmodified Al‑Ge. There are several Σ CSL boundaries in this angular region: Σ5 at 36.86°/<100>, Σ9 at 38.94°/<110>, Σ27a at 31.59°/<110>, and Σ27b at 35.43°/<210>. Clearly, the combination 35°/<110> cannot be assigned to Σ5 because the axes distribution (Supplementary Figure 5a) displays a peak at <110>, not <100>. In practice, a CSL grain boundary can deviate slightly from the ‘correct’ CSL orientation due to dislocations in the lattice. In 1966, Brandon developed a criterion which showed that only boundaries with a maximum deviation $\Delta\theta_{m}$ given by eq. S1 persist as CSL boundaries^2^.

$\Delta\theta_{m}\leq\frac{15^{\circ}}{\sqrt{\Sigma}}$ (S1)

We note that the use of Brandon criterion is a common practice in assigning Σ values, see, *e.g.*, refs.^3,4^ According to the Brandon criterion, the only valid combination for the 35°/<110> angle‑axis pair is Σ9 CSL. Overall, the unmodified and modified samples have similar grain boundary characteristics.

**Supplementary references**

1. DeHoff, R. T., Aigeltinger, E. H. & Craig, K. R. Experimental determination of the topological properties of three-dimensional microstructures. *J. Microsc.* **95,** 69–91 (1972).

2. Brandon, D. G. The Structure of High-Angle Grain Boundaries. *Acta Metall.* **14,** 1479–1484 (1966).

3. Randle, V. The coincidence site lattice and the ‘sigma enigma’. *Mater. Charact.* **47,** 411–416 (2001).

4. Ratanaphan, S., Yoon, Y. & Rohrer, G. S. The five parameter grain boundary character distribution of polycrystalline silicon. *J. Mater. Sci.* **49,** 4938–4945 (2014).
